# Effect of Beta-blockers on Tachycardia in Patients with Pulmonary Embolism

**DOI:** 10.7759/cureus.6512

**Published:** 2019-12-30

**Authors:** Hafiz M Aslam, Hafiz S Naeem, Swati Prabhakar, Talha Awwal, Muhammad Khalid, Anand Kaji

**Affiliations:** 1 Internal Medicine, Seton Hall University / Hackensack Meridian School of Medicine, Trenton, USA; 2 Internal Medicine, St. Francis Medical Center, Trenton, USA

**Keywords:** pulmonary embolism, wells score, geneva score

## Abstract

Hypothesis

Beta-blockers (BBs) lower the heart rate, which may mask the diagnosis of pulmonary embolism (PE) since one of the main clinical diagnoses of PE is tachycardia. The endpoint of our retrospective study is to determine if the pre-existing use of (BB) significantly affects the utility of these scoring criteria in diagnosing PE.

Introduction

Diagnosing PE is a challenge because of the non-specificity of its symptoms and signs. The initial step is to assess the patient’s likelihood of having a PE. This involves using a scoring system to stratify patients into different levels of risk of having PE (for example, as ‘low,’ ‘moderate,’ or ‘high’ risk). Some of the commonly used criteria are Wells’ Score, Geneva Score, and Pulmonary Embolism Rule-out Criteria (PERC) Rule (Charlotte Rule).

Methodology

This retrospective study was conducted at St. Francis Medical Center. Subjects were taken from a patient population with a new diagnosis of PE (between 2010 and 2017) on the basis of computed tomography angiography (CTA) of the chest. Patients with sepsis or septic shock, heart block, atrioventricular (AV) nodal ablation, pacemaker placement, or taking more than one AV nodal blocker were excluded from the study. Subjects were categorized on the basis of beta-blocker consumption.

Result

Out of a total of 170 cases, 71 patients were taking beta-blockers and 99 patients were not taking beta-blockers. Among the participants taking BBs, 30.4% had a heart rate <60 and 55.8% had a heart rate between 60 and 100.

Conclusion

BBs significantly obviate tachycardia in patients with PE. It falsely decreases the Wells’ Score and the Geneva Score and results in the inappropriate fulfilling of PERC criteria.

## Introduction

Acute pulmonary embolism (PE) is a very common and often fatal disease, and, therefore, a quick yet accurate clinical diagnosis is extremely important [1]. Based on the results of a literature search that encompassed all studies indexed by MEDLINE between 1966 and 2014, diagnostic rules of acute PE were developed. The “best practice advice” stated in this report was that “clinicians should use validated clinical prediction rules to estimate pretest probability in patients in whom acute PE is being considered" [2]. On the other hand, lab tests and imaging could be important secondary factors in quick diagnosis [3-4]. This states that clinical judgment is often the most important diagnostic factor in patients presenting with acute PE [5]. Some of the most common presentations of acute PE include dyspnea at rest or with exertion (73%), calf or thigh swelling, erythema, edema, tenderness, palpable cords (47%), and tachycardia (24%) among others [6]. Based on this data, as much as 25% of patients with acute PE will have tachycardia as one of their symptoms. Therefore, the presence of tachycardia is an important diagnostic factor for acute PE. However, patients on beta-blockers (BBs) will have a falsely low heart rate, which, along with other subtle symptoms, could cause confusion and delay in the diagnosis of acute PE. The rapid treatment of PE has been shown to have much better outcomes than delayed treatment [7-8]. Because PE is often underdiagnosed, the disease remains a major health challenge [9].

There are certain criteria that may aid in the diagnosis of PE, namely, the Wells’ Score, the Geneva Score, and the Pulmonary Embolism Rule-out Criteria (PERC) Rule. This study will analyze whether or not BBs alter these scores. These criteria, in addition to the clinical presentation, may aid in the quick diagnosis of PE. There have not been many studies done that show whether or not the Well’s Score, the Geneva Score, and the PERC Rule are meaningful in relation to the mortality rates of patients on BBs presenting with PE. There are studies that aim to evaluate the relationship between BBs and these criteria [10-11].

In this present study, we are hypothesizing that there is a statistically significant difference in heart rates among patients presenting with pulmonary embolism in patients who are taking BBs as compared to those who are not. The study groups will be compared based on their exposure to BBs within the last 24 hours. It is important to study the effect that BBs have on tachycardia in patients with PE to ensure accurate diagnosis, especially in an emergent situation.

The abstract of this article has been presented to the National Meeting of CHEST and American Thoracic Society.

## Materials and methods

Study design

The present study is a nested, retrospective observational study in which there was a review of the charts of the patients admitted to St. Francis Medical Center (SFMC) with a diagnosis of PE.

Subjects

The subjects were identified by a search of diagnostic code and screened for eligibility. There were two groups of subjects: a study group and a control group. The study group consisted of patients diagnosed with PE who had been using BBs at the time of presentation. The control group consisted of patients diagnosed with PE who had not been taking BBs at the time of presentation. The primary endpoint was tachycardia. Secondary analyses included a change in the Wells’ Score, Geneva Score, and PERC Rule. These scoring criteria will be further explained and elaborated on in the discussion section. The objective of this study was to determine the difference in heart rates amongst patients diagnosed with pulmonary embolism who were taking BBs and in patients not taking BBs at the time of presentation to SFMC from 2010 to 2017.

The inclusion criteria for this study included patients with a new diagnosis of PE (diagnosed with a CT angiogram or a ventilation-perfusion scan) and using BBs (exposure/study group) or not using BBs (comparison/control group).

The exclusion criteria for this study included patients with a prior diagnosis of PE, patients who have heart block of any degree, patients with a functioning pacemaker in place, patients taking more than one AV nodal blocker, such as a combination of BBs or a combination of BBs and calcium channel blockers or digoxin, and patients presenting with sepsis or septic shock.

Patients who met the inclusion criteria and were simultaneously not excluded by the exclusion criteria had their personal identifiers, including name, social security number, zip code, and so on, deleted to ensure confidentiality. Each subject was assigned a subject number. All subject data remained at SFMC at all times, and only study investigators involved in the study could access the data.

Statistical analysis

As a retrospective cohort study, we used chart review to assess the patients’ heart rate at the time of presentation to the emergency department (ED). Through data analysis, we determined whether heart rate at the time of presentation significantly differed among subjects taking or not taking BBs for any purpose. We also collected other data, including de-identified demographic baseline information such as sex, age, ethnic origin, education, marital status, social status, immunosuppression status, steroid use, smoking, and other common comorbidities. Other pertinent clinical information was also collected for descriptive purposes and for other possible exploratory analysis. We also took into consideration confounding or intervening variables such as a history of AV nodal ablation or synchronous causes of tachycardia.

All data gathered from charts were directly entered into SPSS 19 software (IBM Corp., Armonk, NY), which allowed for the advanced statistical analysis of the data. Two spreadsheets were used, one for cases and one for controls. Study investigators were the only personnel involved in data collection. Backup files were stored as password-protected files. The password was only released to the principal investigator (PI), co-PI, and co-investigators. Information on the subjects was kept until the end of the analysis. Afterward, the spreadsheets with subject identifiers were deleted as to not violate the Health Insurance Portability and Accountability Act ( HIPAA) laws.

## Results

Out of the qualifying 170 participants, there were 71 patients taking BBs and 99 patients not taking BBs. The data is shown in Table 1. The exact heart rate mean in patients taking BBs was found to be 81.2 with a standard deviation of 24.4, whereas the exact heart rate mean in patients not taking BBs was 96.2 with a standard deviation of 19.2.

**Table 1:** Frequency of patients taking beta-blockers vs. not taking beta-blockers

Beta-Blocker	Number of Patients	Percent of Total Patients
Yes	71	41.8
No	99	58.2
Total:	170	100.0

The exact Wells' Score mean of patients taking BBs was found to be 3.35 with a standard deviation of 2.20, whereas the exact Wells' Score mean of patients not taking BBs was found to be 4.10, with a standard deviation of 2.53. The average Wells' Score of all patients in this study was found to be 3.79 with a standard deviation of 2.42.

There were 29 patients on BBs who had a Wells’ Score of less than two as compared to 24 patients not on BBs who had a Wells’ Score of less than two. There were 18 patients on BBs who had a Wells’ Score of 2-4 as compared to 30 patients not on BBs who had a Wells’ Score of 2-4. There were 21 patients on BBs who had a Wells’ Score of greater than 4 as compared to 45 patients who had a Wells’ Score of greater than 4. The p-value for each of these tests was 0.074. These values are illustrated in Table 2. In patients taking BBs, 42.6% had a Wells’ Score of <2, 26.5% had a score of 2-4, and 30.9% had a score of >4. In patients not taking BBs, 23.2% had a score of <2, 29.3% had a score of 2-4, while 45.5% had a score of >4 (p-value < 0.074) (Table 2).

**Table 2:** Percent of patients categorized by Wells’ Score

Wells’ Score	Number of Patients on Beta-Blockers (% within Wells’ Score)	Number of Patients Not on Beta-Blockers (% within Wells’ Score)	Total (% of total patients):	P-value
<2	29 (54.7)	24 (45.3)	53 (31.7)	< 0.074
2-4	18 (37.5)	30 (62.5)	48 (28.7)	< 0.074
>4	21 (31.8)	45 (68.2)	66 (39.5)	< 0.074
Total:	68 (40.7)	99 (59.3)	167 (100.0)	

The exact Geneva Score mean of patients taking BBs was found to be 4.60, with a standard deviation of 2.60, whereas the exact Geneva Score mean of patients not taking BBs was found to be 6.81 with a standard deviation of 3.22. The average Geneva score of all patients in this study was found to be 5.90 with a standard deviation of 2.42. In patients taking BBs, 44.1% had a Geneva Score of 0-3, 50% had a score of 4-10, and 5.9% had a score of >10. In patients not taking BBs, 16.2% had a score of 0-3, 67.7% had a score of 4-10, and 15.2% had a score of >10 (p-value <0.001) (Table 3).

**Table 3:** Percent of patients categorized by Geneva Score

Geneva Score	Number of Patients on Beta-Blockers (% within Geneva Score)	Number of Patients Not on Beta-Blockers (% within Geneva Score)	Total (% of total patients):	P-value
0-3	30 (65.2)	16 (34.8)	46 (27.5)	0.001
4-10	34 (37.5)	68 (62.5)	102 (61.1)	0.001
>10	4 (21.1)	15 (78.9)	19 (11.4)	0.001
Total:	68 (40.7)	99 (59.3)	167 (100.0)	

There were 30 patients on BBs who had a Geneva Score of 0-3, compared to 16 patients not on BBs who had a Geneva Score of 0-3. There were 34 patients on BBs who had a Geneva Score of 4-10, compared to 68 patients not on BBs who had a Geneva Score of 4-10. There were four patients on BBs who had a Geneva Score of greater than 10 compared to 15 patients who are not on BBs who had a Geneva Score of greater than 10. The p-value for each of these tests was less than 0.01. These values are shown in Table 3.

There were 22 patients on BBs who fulfilled the PERC Rule, compared to four patients not on BBs who fulfilled the PERC Rule. There were 44 patients on BBs who did not fulfill the PERC Rule due to other reasons as compared to 67 patients not on BBs who did not fulfill the PERC Rule due to other reasons. There were two patients on BBs who did not fulfill the PERC Rule due to tachycardia compared to 27 patients not on BBs who did not fulfill the PERC Rule due to tachycardia. The p-value for each of these tests was less than 0.01. These values are shown in Table 4. In patients taking BBs, 32.4% fulfilled the PERC Rule while 64.7% did not fulfill the PERC Rule due to other reasons. In patients not taking BBs, 27.6% did not fulfill the PERC Rule due to tachycardia and 68.4% did not fulfill the PERC Rule due to other reasons (p-value <0.001).

**Table 4:** Percent of patients categorized by PERC criteria PERC: Pulmonary Embolism Rule-out Criteria

PERC Criteria	Number of Patients on Beta-Blockers (% within PERC Criteria)	Number of Patients Not on Beta-Blockers (% within PERC Criteria)	Total (% of total patients):	P-value
Fulfilled	22 (84.6)	4 (15.4)	26 (15.7)	<0.001
Not fulfilled due to other reasons	44 (39.6)	67 (60.4)	111 (66.9)	<0.001
Not fulfilled due to tachycardia	2 (6.9)	27 (93.1)	29 (17.5)	<0.001
Total:	68 (41.0)	98 (59.0)	166 (100.0)	

## Discussion

A research study done in 2008 at Columbia University Medical Center took the data of 130 patients who had developed acute PE between 2005 and 2006. The results showed that patients who had been taking a BB were less likely to be having a tachycardic episode (defined as a heart rate greater than 100 beats per minute) than those who had not been taking a BB. Fifty percent of the patients on BBs had tachycardia on presentation as compared to 69% of the patients not on BBs. The analysis of this data suggests that patients who were taking BBs were less likely to be tachycardic as compared to patients not on BBs [12-13]. This further shows how important it is for physicians to recognize that a patient who does not have tachycardia does not necessarily exclude them from the diagnosis of having an acute PE.

Diagnosing PE is often challenging because the signs and symptoms are not specific [14]. There are various scoring systems that determine whether a patient is more or less likely to have a PE. The most commonly scoring systems include the Wells’ Score, Geneva Score, and PERC Rule.

The Wells’ Score was developed in 1998 and patients are given points based on their history and presentation. Many studies have been done that show the strong efficiency of the Wells’ Score in terms of diagnosing PE [15]. The criteria assessed include signs of deep venous thrombosis (DVT), tachycardia greater than 100 beats per minute, active cancer, and recent immobilization. This gives a patient a possible score ranging from 0 to 12.5. A score greater than 6 indicates that a patient is at “high risk” of having a PE. A score of 2 to 6 indicates “intermediate risk” and a score of less than 2 is classified as “low risk.” Similarly, a two-step Wells’ Score was developed in 2000 to expand on the original Wells’ Score, which takes into consideration the following criteria: clinical signs and symptoms of DVT (score of 3), an alternative diagnosis is less likely than PE (score of 3), a heart rate greater than 100 beats per minute (score of 1.5), immobilization for more than three days or surgery in the previous four weeks (score of 1.5), and previous DVT/PE (score of 1.5), hemoptysis (score of 1), and malignancy, which further includes current treatment, treatment in the last six months, or palliative care (score of 1). Based on these criteria, if the score is less than 4, the clinical probability of PE is low. If the score is higher than 4, the probability of PE is high [16-17].

The Geneva Score is based on seven clinical factors and requires an interpretation of findings on chest X-ray and arterial blood gases [18]. The revised Geneva Score covers eight parameters in three clinical areas, which are risk factors, symptoms, and clinical signs. Each of these is given 1 to 5 points accordingly. This gives a possible score range of 0 to 25. A score of 11 or higher is classified as “high risk” of PE, a score of 4 to 10 is “intermediate risk,” and a score of 0 to 3 is “low risk” [19-20].

The PERC Rule for PE is often used by clinicians especially in the ED environment to rule out pulmonary embolism. It was found in a research study conducted by Siau et al. that the PERC rule is a safe way of excluding PE when used in conjunction with clinical suspicion [12]. The components of the PERC Rule include age greater than 50, heart rate greater than 100, saturation of oxygen at room air less than 95%, unilateral leg swelling, hemoptysis, recent surgery or trauma, prior PE or DVT, or hormone use. If any of these criteria are positive, PE cannot be ruled out based on the PERC rule. BBs are known to obviate tachycardia and can, therefore, theoretically alter inclusion within the PERC Rule [8].

In a meta-analysis of 19 studies (n=25,343), it was found that the diagnosis of acute PE based on clinical impressions alone had a sensitivity and specificity of 85% and 51%, respectively [21]. This shows that in this rapidly progressing disease, it is important for physicians to be able to accurately diagnose the acute PE in order to start treatment immediately. There have not been many studies examining the relationship between the use of BBs and the subsequent diagnosis of acute PE [16].

## Conclusions

In conclusion, this present study is one of the few that has examined how the use of BBs in patients can mask one of the main clinical diagnostic symptoms of acute PE and, therefore, delay the ensuing care, something which could have fatal outcomes. Some limitations in our study include uncontrollable confounding factors in our patients (age, weight, diseases, and other risk factors) as well as not including enough exclusion criteria. Having more studies with perhaps different diagnosis criteria or inclusion/exclusion criteria would help add to the literature. This would ensure the accurate and swift diagnosis of patients who present acutely with this rapidly progressing disease to enhance patient outcomes.
